# Risk factors, clinical features, and outcomes of patients with hypertrophic cardiomyopathy complicated by ischemic stroke: A single-center retrospective study

**DOI:** 10.3389/fcvm.2022.1054199

**Published:** 2022-12-08

**Authors:** Jian-Feng Lu, Ze-Xin Fan, Ying Li, Ze-Yi Wang, Lin Ma, Bo-Yi Yuan, Ting-Ting Yang, Pen-Ju Liu, Guang-Zhi Liu

**Affiliations:** Department of Neurology, Beijing Anzhen Hospital, Capital Medical University, Beijing, China

**Keywords:** hypertrophic cardiomyopathy, ischemic stroke, risk factor, clinical feature, survival

## Abstract

**Objective:**

This study aimed to explore risk factors, clinical features, and prognosis of patients with hypertrophic cardiomyopathy (HCM) complicated by ischemic stroke (IS).

**Methods:**

We conducted a retrospective analysis of all HCM patient data and a 1-year follow-up study.

**Results:**

Totally, 506 patients with HCM, including 71 with IS, were enrolled. Older age (≥63 years) was associated with an increased risk of IS in HCM patients (OR = 1.045, 95% CI: 1.018–1.072, *p* = 0.001). Among 37 patients complicated by IS, 22 (59.5%, 22/37) manifested as cardioembolism (CE) subtype, and 13 (35.1%, 3/37) small artery occlusion (SAO) subtype, according to TOAST classification. In the acute phase, the IS patients presented with NIHSS 4 (interquartile range: 1, 10). Multi-infarction was more common than single infarction (72.7 vs. 27.3%), while cortical + subcortical infarction (CE group: 50%) or subcortical infarction (SAO group: 53.8%) constituted most IS cases. Additionally, the blood supply areas of anterior circulation (CE group: 45.5%; SAO group: 92.3%) or anterior + posterior circulation (CE group: 50%) were mainly involved. The 1-year survival rate of HCM patients with concomitant IS was 81.8%, and IS was associated with 1-year all-cause death in HCM patients (HR = 5.689, 95% CI: 1.784–18.144, *p* = 0.003).

**Conclusion:**

Older age is a risk factor for IS occurrence in HCM patients. Patients with HCM complicated by IS had mild or moderate neurologic deficits at disease onset. CE and SAO subtypes predominate in patients with concomitant IS, especially the former. Multiple cortical and subcortical infarctions are their neuroimaging characteristics, mainly involving the anterior circulation or anterior + posterior circulation. Is is a risk factor for all-cause death in HCM patients within 1 year.

## Introduction

Hypertrophic cardiomyopathy (HCM) is a relatively common, globally distributed, and often hereditary primary heart disease (1), resulting in left ventricular hypertrophy, interstitial fibrosis, impaired ventricular filling, and left ventricular diastolic compliance (2, 3). HCM is a heterogeneous disease with various clinical manifestations, including heart failure (HF), arrhythmias, sudden cardiac death, and thromboembolism (4). Ischemic stroke (IS) is a catastrophic thromboembolic complication of HCM with aging (5, 6), as a previous study reported that the overall incidence of stroke or other vascular events in HCM patients was 0.8%/year and 1.9% for patients >60 years old (7). However, the risk factors, clinical characteristics, and prognosis of HCM complicated with IS remain largely unclear. Hence, we conducted a single-center retrospective study to investigate the risk factors, clinical characterizations of HCM-IS, and a 1-year follow-up to examine the short-term survival rate.

## Materials and methods

### Study population

From May 2019 to May 2021, 629 HCM inpatients from Beijing Anzhen Hospital of Capital Medical University were selected. The following inclusion criteria were used: (1) ≥18 years; (2) HCM diagnosis according to the guidelines of the American College of Cardiology Foundation (ACCF) and the American Heart Association (AHA) based on hypertrophic and non-dilated cardiomyopathy confirmed by echocardiography or cardiac magnetic resonance imaging (MRI), and exclusion of other cardiac or systemic diseases (8). The exclusion criteria were (1) life expectancy ≤ 1 year, (2) patients unable to cooperate with follow-up, and (3) patients with incomplete data. Patients with IS were diagnosed based on their medical history, clinical examination, and the results of cranial MRI and magnetic resonance angiography scans confirmed by two attending neurologists. All participants signed written informed consent before the start of the study, which was approved by the Ethical Committee of Beijing Anzhen Hospital.

### Clinical data extraction

All clinical data were collected from the electronic medical record system at the time of first hospital admission, including demographics, past medical history, echocardiography and laboratory examinations, drug use, and treatment.

### One-year follow-up

Trained investigators followed up with all patients *via* face-to-face interviews or phone calls for at least 1 year. The main outcome was all-cause death, including cardiogenic death during hospitalization or 1 year after onset, IS complications, or death from other causes.

### Statistics

Statistical analyses were performed using SPSS (version 26.0; SPSS, Chicago, IL, USA). Continuous variables are presented as mean ± standard deviation or median (interquartile range [IQR]). Categorical variables were expressed as percentages. Normally distributed data were analyzed using Student's *t*-test, and non-normally distributed data were analyzed using Mann–Whitney *U* test. Categorical variables were analyzed using chi-squared test. Multivariate logistic regression analyses were utilized to screen out the risk factors associated with HCM complicated by IS. The groups were compared based on whether HCM patients had all-cause deaths within 1 year, and then the statistically different variables in the comparison between groups were included in the multivariate Cox proportional hazard model. Survival curves were estimated using the Kaplan–Meier method and compared with the log-rank test. Statistical significance was set at *p* < 0.05.

## Results

### Patient selection

**Figure 1:**
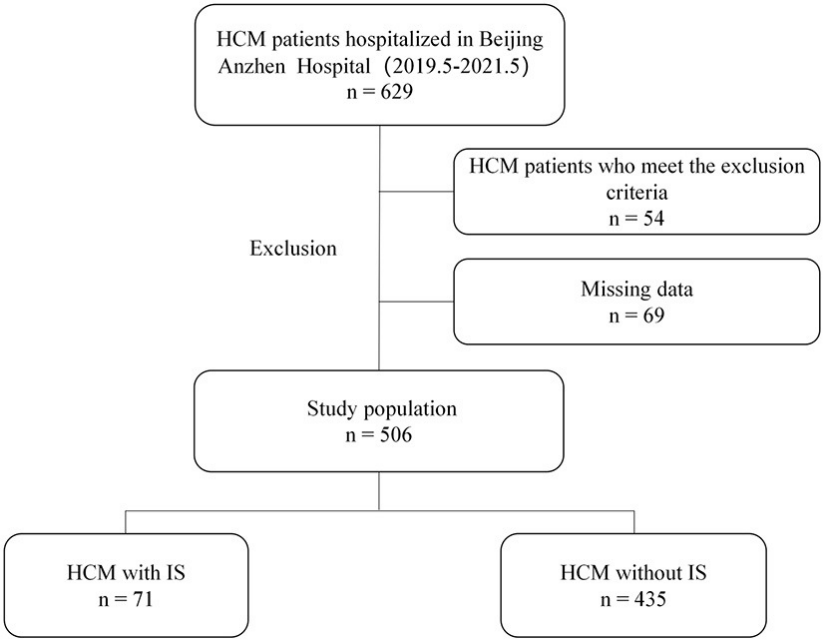
Flowchart describing the enrollment of patients with hypertrophic cardiomyopathy (HCM) complicated by ischemic stroke (IS).

Among 629 patients with HCM, 506 (298 males and 208 females) were eligible for this study, including 71 with IS and 435 without IS. The flowchart for patients' selection in the study is revealed in Figure 1.

### Risk factors of HCM complicated with ischemic stroke

Multiple variables, such as basic characteristics, stroke risk factors, electrocardiogram, echocardiography (i.e., left ventricular end-diastolic diameter, left ventricular ejection fraction, left atrial diameter, and intracardiac thrombus), and laboratory results, were compared between patients with and without IS (Table 1). As a result, there were marked differences between these two groups in terms of age (≥63 years), type 2 diabetes, estimated glomerular filtration rate (eGFR), and D-dimer (Table 1). Four significant variables were retained in the final multivariate logistic regression model, followed by enter variable selection. Age was associated with an increased risk of IS in HCM patients (OR = 1.045, 95% CI: 1.018–1.072, *p* = 0.001) (Table 2).

**Table 1:** Comparison of baseline data in patients with hypertrophic cardiomyopathy (HCM) complicated with or without ischemic stroke (IS).

**Category**	**HCM with IS (*n* = 71)**	**HCM w/o IS (*n* = 435)**	***p*-value**
Age	63 (58, 70)	57 (48, 65)	**< 0.001**
Male	40 (56.3%)	258 (59.3%)	0.637
Smoking	14 (19.7%)	111 (25.5%)	0.294
Drinking	12 (16.9%)	84 (19.3%)	0.631
Gout	5 (7.0%)	27 (6.2%)	0.789
Hypertension	40 (56.3%)	197 (45.3%)	0.084
Hyperlipidemia	32 (45.1%)	153 (35.2%)	0.108
Type 2 diabetes	18 (25.4%)	63 (14.9%)	**0.021**
AF	21 (29.6%)	106 (24.4%)	0.348
Cardiac function grade III-IV	9 (12.7%)	61 (14.0%)	0.761
Left bundle branch block	11 (15.5%)	85 (19.5%)	0.420
Moderate to severe mitral regurgitation	7 (9.9%)	76 (17.5%)	0.108
Leucocyte (10^9^/L)	6.4 (5.7, 8.4)	6.9 (5.8, 8.4)	0.111
Platelet (10^9^/L)	202 (148, 232)	198 (156, 236)	0.916
Hemoglobin (g/L)	138 (124, 152)	143 (126,153)	0.223
eGFR (ml/min/1.73 m^2^)	88.5 (77.5, 98.0)	95.7 (85.2, 104.5)	**0.001**
Blood sodium (mmol/L)	140.2 (138.3, 141.9)	140.8 (139.2, 142.2)	0.168
BNP	509 (197, 606)	376 (177, 628)	0.152
Hs-CRP (mg/L)	0.95 (0.58, 2.44)	0.98 (0.65, 1.84)	0.813
D-Dimer (ng/ml)	134 (63, 258)	98 (64, 174)	**0.015**
Echocardiography			
LVEDD	45 (41, 48)	45 (41, 48)	0.713
LVEF	61 (56, 66)	64 (58, 68)	0.052
LAD	41 (38, 46)	41 (39, 45)	0.736
Intracardiac thrombus	1 (1.4%)	2 (0.5%)	0.334

AF, atrial fibrillation; eGFR, estimated glomerular filtration rate; Hs-CRP, hypersensitive C-reactive protein; LVEDD, left ventricular end-diastolic diameter; LVEF, left ventricular ejection fraction; LAD, left atrial diameter. Bold values indicate *p* < 0.05.

**Table 2:** Risk factors for ischemic stroke (IS) in patients with hypertrophic cardiomyopathy (HCM).

**Group**	**HCM with IS (*n* = 71)**	**HCM w/o IS (*n* = 435)**	**B**	**SE**	**Wald**	**OR (95% CI)**	***p*-value**
Type 2 diabetes	18 (25.4%)	63 (14.9%)	0.358	0.313	1.307	1.430 (0.774, 2.642)	0.253
Age (≥63 years)	63 (58, 70)	57 (48, 65)	0.044	0.013	11.094	1.045 (1.018, 1.072)	**0.001**
eGFR (ml/min/1.73 m^2^)	88.5 (77.5, 98.0)	95.7 (85.2, 104.5)	−0.009	0.007	1.568	0.991 (0.977, 1.005)	0.210
D-Dimer (ng/ml)	134 (63, 258)	98 (64, 174)	0.000	0.000	0.116	1.000 (1.000, 1.000)	0.733

eGFR, estimated glomerular filtration rate. Bold values indicate *p* < 0.05.

### Clinical features and brain imaging

Among 37 HCM patients complicated by IS, two (5.4%, 2/37) were categorized as large vessel atherosclerosis (LAA) subtype, 22 (59.5%, 22/37) as cardioembolism (CE) subtype, and 13 as small artery occlusion (SAO) subtype (35.1%, 13/37) according to TOAST classification. In the acute phase, the IS patients presented with relatively mild or moderate clinical deficits, as reflected by the NIHSS scores (4, interquartile range: 1, 10). Multi-infarction is more common than single infarction (72.7 vs. 27.3%). Most cases had cortical + subcortical infarction (cardiogenic embolism group: 50%) or subcortical infarction (SAO group: 53.8%) mainly involving anterior circulation (CE group: 45.5%; SAO group: 92.3%) or anterior + posterior circulation (CE group: 50%) (Table 3).

**Table 3:** Brain MRI features of hypertrophic cardiomyopathy with ischemic stroke (*n* = 37).

**Group**	**CE**	**SAO**	**LAA**
	**(*n* = 22)**	**(*n* = 13)**	**(*n* = 2)**
Single infarct			
Cortical	1	6	1
Subcortical	3	0	0
brainstem	2	0	1
Multiple infarcts			
Cortical	4	0	0
Cortical+subcortical	11	0	0
subcortical	1	7	0
Vessel localization			
anterior	10	12	1
Posterior	1	1	1
Anterior+posterior	11	0	0

CE, cardioembolism; LAA, large vessel atherosclerosis; SAO, small artery occlusion.

### One-year survival

**Figure 2:**
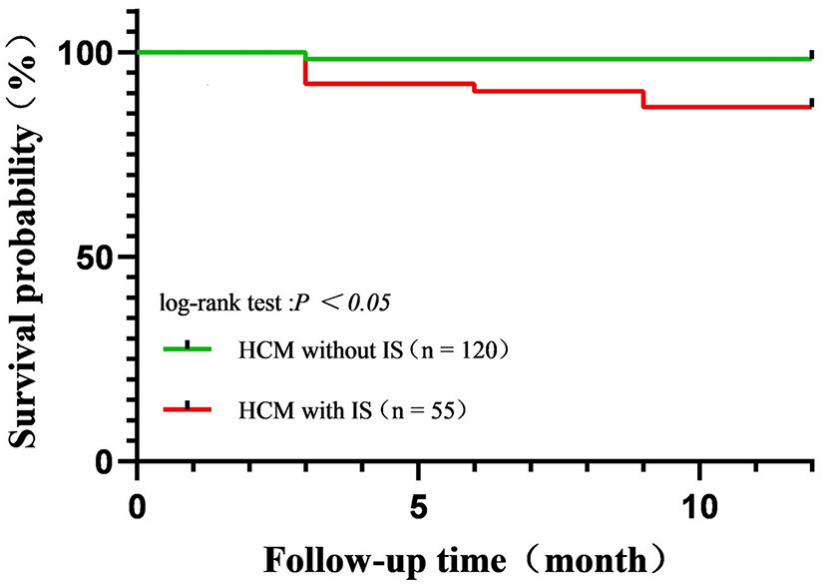
Kaplan–Meier curves for survival rate in patients with hypertrophic cardiomyopathy complicated by ischemic stroke (IS) and HCM complicated without IS. Overall, the 1-year survival rate was 81.8% in patients with HCM with IS and 96.7% in patients with HCM without IS.

Among 506 HCM patients, 175 (55 HCM patients with IS and 120 HCM patients without IS) were followed up for at least 1 year, with a median time of 15 (IQR: 12, 20) months. During the follow-up period, stroke recurred in five patients with HCM complicated by IS (5/55, 9.1%). Death was recorded in 14 patients (10 cases of HCM with IS, four cases of HCM without IS), with overall 1-year survival rates of 81.8% in HCM patients with IS and 96.7% in HCM patients without IS (Figure 2). The cumulative survival rate differed significantly between these two groups (log-rank *p* = 0.016). Causes of death included HF (*n* = 5), acute coronary syndrome (*n* = 6), massive cerebral infarction (*n* = 2), and unknown etiology (*n* = 1).

In univariate analyses, four baseline variables and IS were associated with all-cause death, but only IS (HR = 5.689, 95% CI: 1.784–18.144, *p* = 0.003) was retained in the final multivariate Cox proportional hazard model (Table 4).

**Table 4:** Related factors of 1-year all-cause death in patients with hypertrophic cardiomyopathy (*n* = 175).

	**Univariate analysis**		**Multivariate analysis**	
	**HR (95% CI)**	***p-*value**	**HR (95% CI)**	***p-*value**
IS	5.689 (1.784–18.144)	0.003	5.689 (1.784–18.144)	**0.003**
eGFR (ml/min/1.73 m^2^)	0.971 (0.949–0.994)	0.015		0.051
Hemoglobin	0.978 (0.961–0.996)	0.015		0.064
Hs-CRP	1.168 (1.063–1.283)	0.047		0.104
Age	1.058 (1.007–1.112)	0.025		0.157

eGFR, estimated glomerular filtration rate; HR, hazard ratio; Hs-CRP, hypersensitive C-reactive protein; IS, ischemic stroke. Bold values indicate *p* < 0.05.

## Discussion

As a catastrophic adverse event, thromboembolism events (stroke and systemic embolic events) have a high incidence or prevalence in HCM patients worldwide, eventually contributing to increased morbidity and mortality (4, 9–12). To date, several risk factors for thromboembolism have been confirmed in HCM patients, including atrial fibrillation (AF), age, left atrial diameter, and HF. In the present study, we found that among all HCM patients, there were significant differences in age (≥63 years), type 2 diabetes, eGFR, and D-dimer levels between patients complicated with IS and those without IS. Furthermore, a multiple regression analysis revealed that age was an independent risk factor for the occurrence of IS in HCM. Consistent with the findings of two distinct retrospective studies (7, 13), our results indicate that a higher risk of IS is associated with older age in HCM patients. The explanation for this is that the abnormal morphology and function of the left atrial appendage render HCM patients prone to developing thromboembolic events since elderly patients have a higher incidence of abnormal cardiac structure and function (14). Additionally, as age increases, other cardiogenic or non-cardiogenic complications, such as hypertension, hyperlipidemia, diabetes, deep vein thrombosis, and AF, may increase the incidence of IS and affect long-term stroke outcomes in HCM patients (15–18). Hence, risk factor screening and early intervention should be performed routinely on elderly patients with HCM to prevent the occurrence of IS.

AF is the most common arrhythmia in HCM patients and is associated with a high risk of thromboembolism (19). In a Korean population–based study, the prevalence of stroke among HCM patients with AF was 20%, two times higher than the prevalence in all HCM patients (9). However, our study found no obvious association between AF and the risk of IS in HCM patients, possibly due to the relatively small number of HCM patients with AF. Interestingly, a recent large-scale study on HCM patients without AF (*n* = 17,371) found that their risk of IS was similar to that of controls from the general population with AF taking oral anticoagulants, indicating that HCM could carry a similarly high risk of thromboembolism as AF. The plausible explanation is that aging and other cardiovascular diseases can cause atrial cardiomyopathy, which in turn causes AF and thromboembolism (20). Evidently, our study demonstrated a significant association of older age with the higher risk of IS in HCM patients, mainly in the absence of AF. Nevertheless, more evidence is needed to address this issue in the future.

As one of the common complications of HCM, the risk of thromboembolism (IS and systemic embolism) is high in HCM patients mainly due to AF, as well as older age and left atrial dilatation (11, 21, 22). In our study, according to the TOAST classification, CE and SAO stroke constituted most HCM cases, particularly the former, revalidating that thromboembolism underlies the pathogenesis of IS. Notably, for the first time, our study reported brain neuroimaging characteristics of patients with HCM complicated by IS, such as multiple infarcts distributed in the cerebral cortex and subcortical region, as well as multi-vessel involvement, almost in line with the neuroimaging profile of cardiogenic stroke (23). However, considering the small sample size, further studies with larger sample sizes should be performed to confirm our results.

Previous studies have reported a high annual mortality rate of 3–6% among HCM patients worldwide (24–27), and the leading cause of death included sudden death and HF complications (28, 29). With the advancement of treatment methods over the past few decades, the prognosis for HCM has greatly improved, with an overall annual mortality rate of < 1% (13, 30–34). In the present study, the mortality rate for HCM patients was 8% during a 1-year follow-up period, and progressive HF and acute coronary syndrome were the leading causes of death. Compared to patients without IS, the proportion of adverse outcomes in patients with IS was relatively higher, implying that IS is the main cause of the increased mortality rate of HCM. Furthermore, Cox multivariate analysis revealed that IS, as a complication of HCM, was an independent risk factor for all-cause death in HCM patients, thus reconfirming our viewpoint to a large extent. Consistent with this, several studies have reported that stroke and peripheral embolism, two severe complications of HCM, may cause more disability and death in the elderly, particularly those over the age of 60 (7, 15). Hence, a detailed clinical evaluation and timely intervention should be conducted in HCM patients complicated with IS, especially the elderly, to improve the prognosis of HCM as much as possible.

The role of inflammation in IS has drawn great attention since neuronal death caused by occlusion of cerebral blood flow can trigger both local and systemic immune responses (35, 36). Accordingly, individuals with signs of inflammation or corresponding biomarkers have an increased risk of stroke (37). Of them, atherosclerosis, a well-known chronic inflammatory disease, can cause heart attack and ischemic stroke in the advanced stage (38). Notably, increased aortic pulse wave velocity (aPWV), an independent cardiovascular risk factor representing aortic stiffness, was found to be associated with inflammation (39), thereby indicating the potential clinical use of the measure of aPWV in IS. Nevertheless, the exact relationship between this parameter and HCM complicated with IS needs to be further ascertained.

In conclusion, we found that in HCM patients with IS, an older age (≥63 years) is a risk factor for the occurrence of stroke. In the classification of TOAST, CE and SAO subtypes predominate in patients with concomitant IS, especially the former. The IS patients had mild or moderate neurologic deficit at disease onset. Multiple, cortical, and subcortical infarctions are their neuroimaging characteristics, mainly involving the anterior circulation or anterior + posterior circulation. HCM patients complicated by IS have good short-term survival, and IS is a risk factor for all-cause deaths among HCM patients within 1 year. These findings will help neurologists and cardiologists identify HCM patients at high risk for IS and prevent its occurrence. However, our study has some limitations. First, because this was a single-center retrospective study, a significant bias could be produced by the incomplete clinical and imaging data (cardiac MRI, clinical evaluation after treatment, etc.); Second, an obvious limitation of this study is its lack of statistical power due to the small sample size, so the findings should be interpreted with high caution. Finally, the follow-up time was relatively short for most patients. Therefore, future multi-center, prospective studies using a larger sample size will help validate our findings' reliability and provide valuable new evidence for preventing and treating IS in HCM patients.

## Data availability statement

The original contributions presented in the study are included in the article/supplementary material, further inquiries can be directed to the corresponding author.

## Ethics statement

The studies involving human participants were reviewed and approved by Research Ethical Committee of Beijing Anzhen Hospital. The patients/participants provided their written informed consent to participate in this study.

## Author contributions

G-ZL conceived the experiments, J-FL, Z-XF, and YL conducted the experiments. Z-YW, LM, B-YY, T-TY, and P-JL analysed the results. All authors contributed to the article and approved the submitted version.

## References

[1] Maron BJ Desai MY Nishimura RA Spirito P Rakowski H Towbin JA. Management of hypertrophic cardiomyopathy: JACC state-of-the-art review. J Am Coll Cardiol. (2022) 79:390–414. 10.1016/j.jacc.2021.11.021 35086661

[2] Tuohy CV Kaul S Song HK Nazer B Heitner SB. Hypertrophic cardiomyopathy: the future of treatment. Eur J Heart Fail. (2020) 22:228–40. 10.1002/ejhf.1715 31919938

[3] Marian AJ Braunwald E. Hypertrophic cardiomyopathy: genetics, pathogenesis, clinical manifestations, diagnosis, and therapy. Circ Res. (2017) 121:749–70. 10.1161/CIRCRESAHA.117.311059 28912181 PMC5654557

[4] Liu L Liu Z Chen X He S. Thromboembolism in patients with hypertrophic cardiomyopathy. Int J Med Sci. (2021) 18:727–35. 10.7150/ijms.50167 33437207 PMC7797548

[5] Mizia-Stec K Caforio ALP Charron P Gimeno JR Elliott P Kaski JP. Atrial fibrillation, anticoagulation management and risk of stroke in the Cardiomyopathy/Myocarditis registry of the EURObservational Research Programme of the European Society of Cardiology. ESC Heart Fail. (2020) 7:3601–9. 10.1002/ehf2.12854 32940421 PMC7754739

[6] Finsterer J Stöllberger C Wahbi K. Cardiomyopathy in neurological disorders. Cardiovasc Pathol. (2013) 22:389–400. 10.1016/j.carpath.2012.12.008 23433859

[7] Maron BJ Olivotto I Bellone P Conte MR Cecchi F Flygenring BP. Clinical profile of stroke in 900 patients with hypertrophic cardiomyopathy. J Am Coll Cardiol. (2002) 39:301–7. 10.1016/S0735-1097(01)01727-2 11788223

[8] Gersh BJ Maron BJ Bonow RO Dearani JA Fifer MA Link MS. 2011 ACCF/AHA guideline for the diagnosis and treatment of hypertrophic cardiomyopathy: executive summary: a report of the American College of Cardiology Foundation/American Heart Association Task Force on Practice Guidelines. Circulation. (2011) 124:2761–96. 10.1161/CIR.0b013e318223e230 22068435

[9] Choi YJ Choi EK Han KD Jung JH Park J Lee E. Temporal trends of the prevalence and incidence of atrial fibrillation and stroke among Asian patients with hypertrophic cardiomyopathy: a nationwide population-based study. Int J Cardiol. (2018) 273:130–5. 10.1016/j.ijcard.2018.08.038 30150122

[10] Hirota T Kubo T Baba Y Ochi Y Takahashi A Yamasaki N. Clinical profile of thromboembolic events in patients with hypertrophic cardiomyopathy in a regional japanese cohort —results from kochi RYOMA study. Circ J. (2019) 83:1747–54. 10.1253/circj.CJ-19-0186 31257313

[11] Guttmann OP Pavlou M O'Mahony C Monserrat L Anastasakis A Rapezzi C. Prediction of thrombo-embolic risk in patients with hypertrophic cardiomyopathy (HCM Risk-CVA). Eur J Heart Fail. (2015) 8:837–45. 10.1002/ejhf.316 26183688 PMC4737264

[12] Lorenzini M Anastasiou Z O'Mahony C Guttman OP Gimeno JR Monserrat L. Mortality among referral patients with hypertrophic cardiomyopathy vs the general european population. JAMA Cardiol. (2020) 5:73–80. 10.1001/jamacardio.2019.4534 31774458 PMC6902239

[13] Maron BJ Olivotto I Spirito P Casey SA Bellone P Gohman TE. Epidemiology of hypertrophic cardiomyopathy-related death: revisited in a large non-referral based patient population. Circulation. (2000) 102:858–64. 10.1161/01.CIR.102.8.858 10952953

[14] Philipson DJ Rader F Siegel RJ. Risk factors for atrial fibrillation in hypertrophic cardiomyopathy. Eur J Prev Cardiol. (2019) 2019:2047487319828474. 10.1177/2047487319828474 30727760

[15] Maron BJ Rowin EJ Casey SA Haas TS Chan RH Udelson JE. Risk stratification and outcome of patients with hypertrophic cardiomyopathy >=60 years of age. Circulation. (2013) 127:585–93. 10.1161/CIRCULATIONAHA.112.136085 23275385

[16] Pinto A Di Raimondo D Tuttolomondo A Fernandez P Arnao V Licata G. Twenty-four hour ambulatory blood pressure monitoring to evaluate effects on blood pressure of physical activity in hypertensive patients. Clin J Sport Med. (2006) 16:238–43. 10.1097/00042752-200605000-00009 16778545

[17] Lensing AW. Anticoagulation in acute ischaemic stroke: deep vein thrombosis prevention and long-term stroke outcomes. Blood Coagul Fibrinolysis. (1999) 10(Suppl 2):S123–7. 10493241

[18] Siragusa S Malato A Saccullo G Iorio A Di Ianni M Caracciolo C. Residual vein thrombosis for assessing duration of anticoagulation after unprovoked deep vein thrombosis of the lower limbs: the extended DACUS study. Am J Hematol. (2011) 86:914–7. 10.1002/ajh.22156 21953853

[19] Guttmann OP Rahman MS O'Mahony C Anastasakis A Elliott PM. Atrial fibrillation and thromboembolism in patients with hypertrophic cardiomyopathy: systematic review. Heart. (2014) 100:465–72. 10.1136/heartjnl-2013-304276 24014282

[20] Lin TT Sung YL Ko TY Lee CK Lin LY Juang JJ. Risk of ischemic stroke in patients with hypertrophic cardiomyopathy in the absence of atrial fibrillation - a nationwide cohort study. Aging (Albany NY). (2019) 11:11347–57. 10.18632/aging.102532 31794426 PMC6932926

[21] Nasser MF Gandhi S Siegel RJ Rader F. Anticoagulation for stroke prevention in patients with hypertrophic cardiomyopathy and atrial fibrillation: a review. Heart Rhythm. (2021) 2:297–302. 10.1016/j.hrthm.2020.09.018 33022393

[22] Haruki S Minami Y Hagiwara N. Stroke and embolic events in hypertrophic cardiomyopathy: risk stratification in patients without atrial fibrillation. Stroke. (2016) 47:936–42. 10.1161/STROKEAHA.115.012130 26941260

[23] Liu GZ Hu R Peng DT. Geriatric Neurology Group, Geriatric Branch of Chinese Medical Association; Writing Group of Chinese expert consensus on diagnosis of cardiogenic stroke. Chinese expert consensus on the diagnosis of cardiogenic stroke. Chin Med J (Engl). (2019) 134:505–7. 10.1097/CM9.0000000000001217 33652457 PMC7929520

[24] Maron BJ Spirito P. Impact of patient selection biases on the perception of hypertrophic cardiomyopathy and its natural history. Am J Cardiol. (1993) 72:970–2. 10.1016/0002-9149(93)91117-Z 8213558

[25] Braunwald E Lambrew CT Rockoff SD Ross J Morrow AG. Idiopathic hypertrophic subaortic stenosis: a description of the disease based upon an analysis of 64 patients. Circulation. (1964) 30:3–119. 10.1161/01.CIR.29.5S4.IV-3 14227306

[26] Hardarson T De la Calzada CS Curiel R Goodwin JF. Prognosis and mortality of hypertrophic obstructive cardiomyopathy. Lancet. (1973) 2:1462–7. 10.1016/S0140-6736(73)92730-X 4129311

[27] Shah PM Adelman AG Wigle ED Gobel FL Burchell HB Hardarson T. The natural (and unnatural) history of hypertrophic obstructive cardiomyopathy. Circ Res. (1974) 35:179–95. 4152327

[28] Maron BJ. Clinical course and management of hypertrophic cardiomyopathy. Engl N J Med. (2018) 379:655–68. 10.1056/NEJMra1710575 30110588

[29] Maron BJ Ommen SR Semsarian C Spirito P Olivotto I Maron MS. Hypertrophic cardiomyopathy: present and future, with translation into contemporary cardiovascular medicine. J Am Coll Cardiol. (2014) 64:83–99. 10.1016/j.jacc.2014.05.003 24998133

[30] Shapiro LM Zezulka A. Hypertrophic cardiomyopathy: a common disease with a good prognosis. Five year experience of a district general hospital. Br Heart J. (1983) 50:530–3. 10.1136/hrt.50.6.530 6686058 PMC481455

[31] Spirito P Chiarella F Carratino L Berisso MZ Bellotti P Vecchio C. Clinical course and prognosis of hypertrophic cardiomyopathy in an outpatient population. N Engl J Med. (1989) 320:749–55. 10.1056/NEJM198903233201201 2646539

[32] Cecchi F Olivotto I Montereggi A Gennaro S Alberto M Barry JM. Hypertrophic cardiomyopathy in Tuscany: clinical course and outcome in an unselected regional population. J Am Coll Cardiol. (1995) 26:1529–36. 10.1016/0735-1097(95)00353-3 7594081

[33] Maron BJ Casey SA Poliac LC Gohman TE Almquist AK Aeppli DM. Clinical course of hypertrophic cardiomyopathy in a regional United States cohort. JAMA. (1999) 281:650–5. 10.1001/jama.281.7.650 10029128

[34] Maron BJ Rowin EJ Casey SA Link MS Lesser JR Chan RH. Hypertrophic cardiomyopathy in adulthood associated with low cardiovascular mortality with contemporary management strategies. J Am Coll Cardiol. (2015) 65:1915–28. 10.1016/j.jacc.2015.02.061 25953744

[35] Maida CD Norrito RL Daidone M Tuttolomondo A Pinto A. Neuroinflammatory mechanisms in ischemic stroke: focus on cardioembolic stroke, background, and therapeutic approaches. Int J Mol Sci. (2020) 21:6454. 10.3390/ijms21186454 32899616 PMC7555650

[36] Muhammad S Chaudhry SR Kahlert UD Niemelä M Hänggi D. Brain immune interactions-novel emerging options to treat acute ischemic brain injury. Cells. (2021) 10:2429. 10.3390/cells10092429 34572077 PMC8472028

[37] Endres M Moro MA Nolte CH Dames C Buckwalter MS Meisel A. Immune Pathways in etiology, acute phase, and chronic sequelae of ischemic stroke. Circ Res. (2022) 130:1167–86. 10.1161/CIRCRESAHA.121.319994 35420915

[38] Taleb S. Inflammation in atherosclerosis. Arch Cardiovasc Dis. (2016) 109:708–15. 10.1016/j.acvd.2016.04.002 27595467

[39] Zanoli L Boutouyrie P Fatuzzo P Granata A Lentini P Oztürk K. Inflammation and aortic stiffness: an individual participant data meta-analysis in patients with inflammatory bowel disease. J Am Heart Assoc. (2017) 6:e007003. 10.1161/JAHA.117.007003 29018026 PMC5721883

